# Supplementary material for: Regulation of Satiety by Bdnf-e2-Expressing Neurons through TrkB Activation in Ventromedial Hypothalamus
Source: Biomolecules. 2023 May 11;13(5):822. doi: 10.3390/biom13050822 (PMC10216274; doi:10.3390/biom13050822)
Supplement: Supplementary file 1 [file biomolecules-13-00822-s001.zip › biomolecules-2334514-supplementary.pdf]

Supplementary Materials

Table S1. Repeat experiments of 12-day food intake in *Bdnf-e1*$^{-/-}$ mice.

Repeat number	Age (month)	Daily food intake (g)		p value[#]
		Bdnf-e1$^{-/-}$	WT littermates	
1[*]	5	4.97±0.14	4.81±0.24	0.5464
2	4	4.01±0.29	3.68±0.10	0.2646

* This repeat experiment was performed by a independent CRO. # Data were presented as mean ± SEM. p value for RM two-way ANOVA.

Figure S1

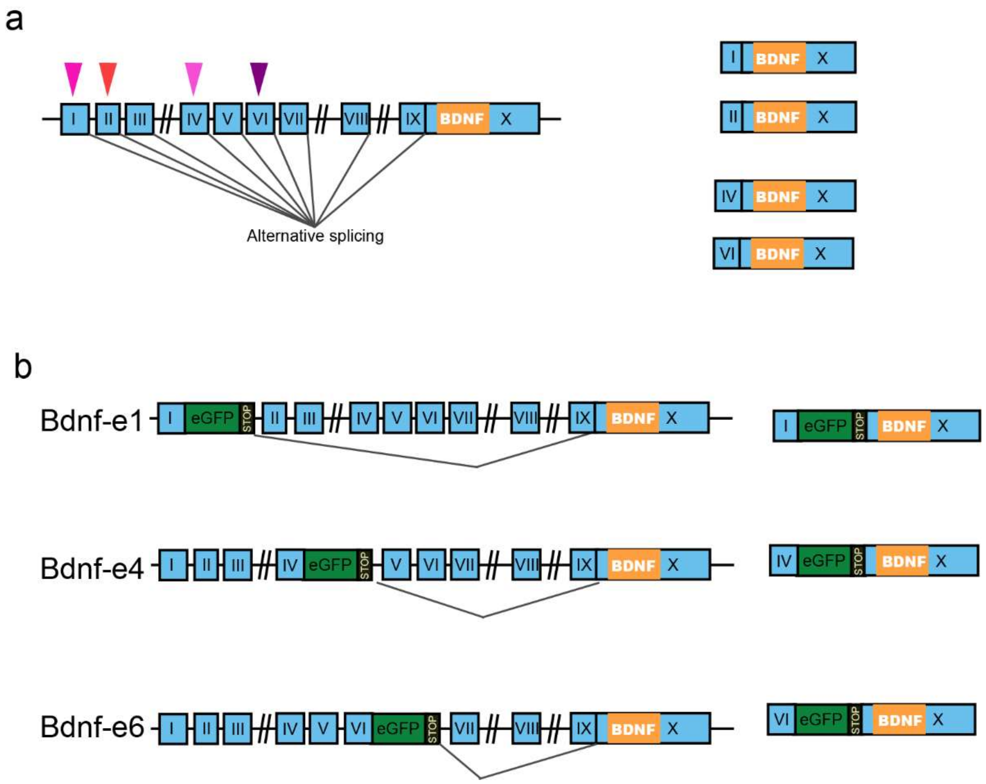

Figure S1: Schematic diagrams of WT and mutant *Bdnf* gene.
(a) Schematic diagrams for the genomic structure of *Bdnf* and four transcripts driven by promoter I, II, IV and VI. **(b)** Schematic diagrams of splicing and the consequent transcripts in *Bdnf*-e1$^{-/-}$, *Bdnf*-e4$^{-/-}$ and *Bdnf*-e6$^{-/-}$ mice.

Figure S2

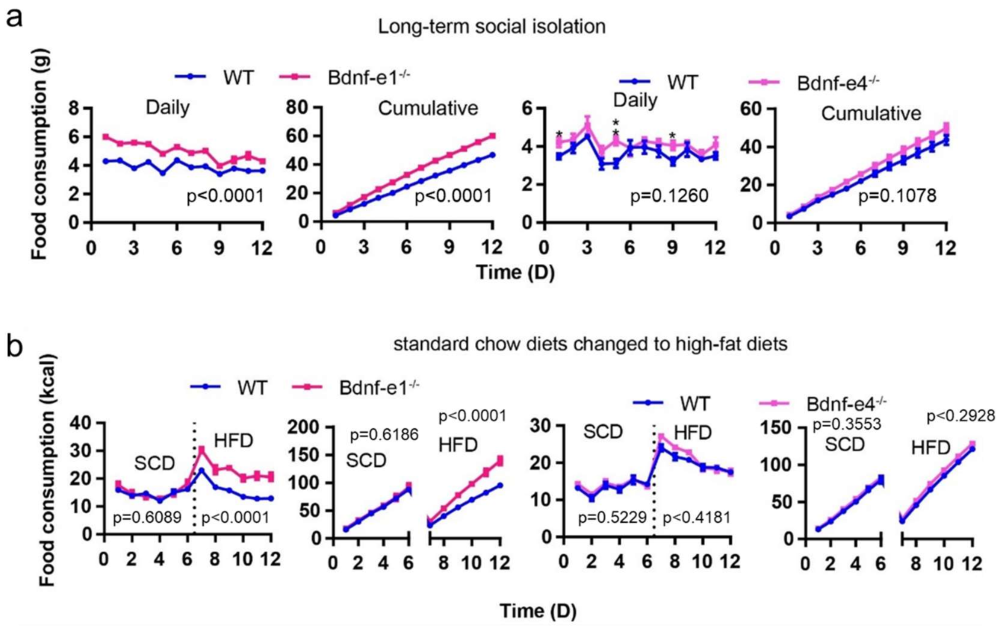

Figure S2: Food intake analysis under the stress of social isolation or high-fat diets. **(a)** Daily and cumulative food consumption of *Bdnf*-e1$^{-/-}$, *Bdnf*-e4$^{-/-}$ and WT littermates, after long-term social isolation for more than one month. **(b)** Daily and cumulative food consumption of *Bdnf*-e1$^{-/-}$, *Bdnf*-e4$^{-/-}$ and WT littermates, when food was changed from standard chow diets (SCD) to high-fat diets (HFD). p values of two-way RM ANOVA for daily and cumulative data were labeled correspondingly in panels.

Figure S3

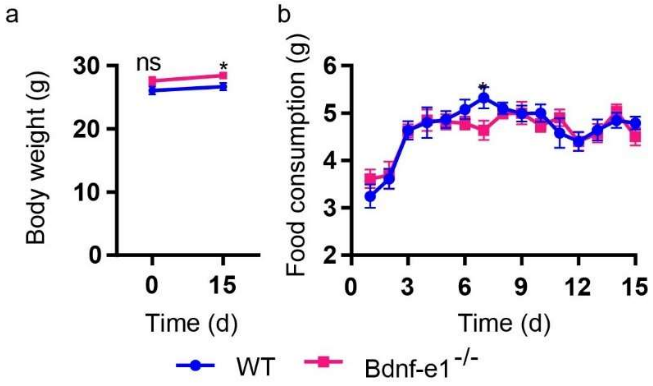

Figure S3: Food consumption in *Bdnf-e1*$^{-/-}$ mice.
(a) Food consumption and body weight of *Bdnf*-e1$^{-/-}$ mice and their WT littermates during the onset of obesity (n=8~9/group). **(b)** Food consumption f *Bdnf*-e6$^{-/-}$ mice and their WT littermates (n=5~6/group).

Figure S4

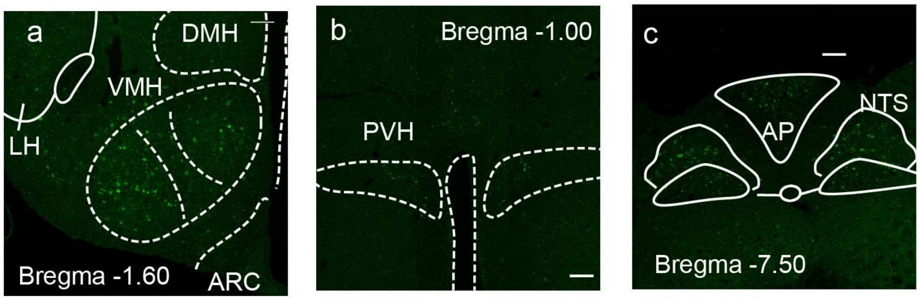

Figure S4: Large images of pII-driven GFP immunostaining.
(a,b) Hypothalamus and **(c)** DVC. Scale bar: 50 μm.

Figure S5

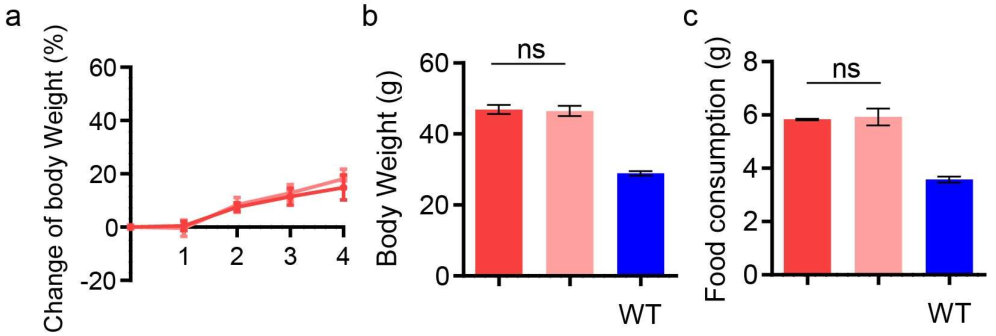

Figure S5: Injection of AAV- e2-BDNF-Myc into DVC.
(a) Change of body weight after injection of AAVs into DVC in *Bdnf*-e2$^{-/-}$ mice (n=3~4/group). p value of two-way RM ANOVA was 0.7783. **(b)** Body weight of *Bdnf*-e2-/- mice 4 weeks with injection of AAVs into DVC (n=3~4/group), compared with WT littermates (n=7). **(c)** Average daily food intake of *Bdnf*-e2-/- mice 4 weeks with injection of AAVs into DVC (n=3~4/group), compared with WT littermates (n=7).

Figure S6

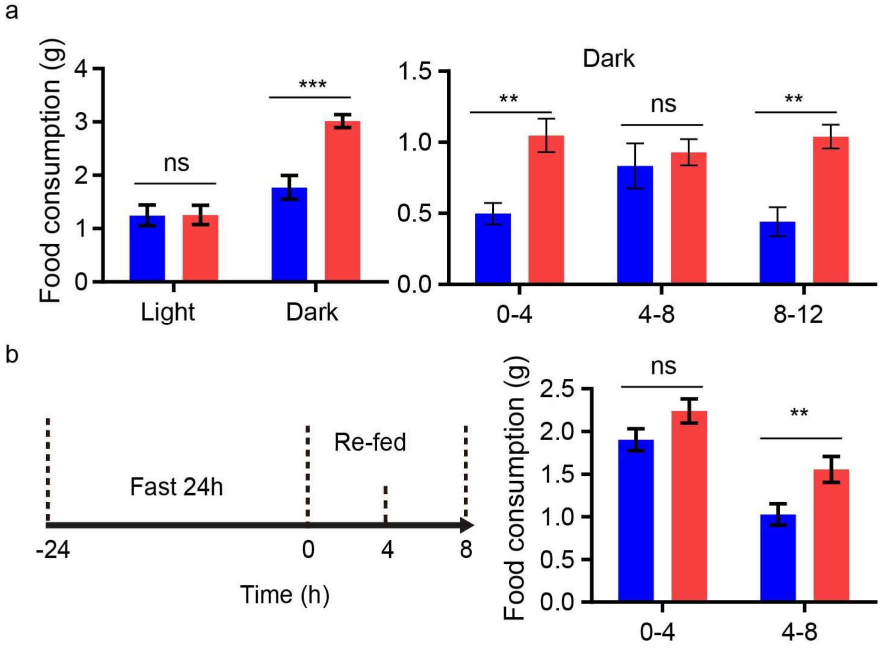

Figure S6: Less satiety in *Bdnf-e2*$^{-/-}$ mice.
(a) Food consumption during different periods. Numbers in the bottom indicate 0-4, 4-8, 8-12 hours during darkness. **(b)** Food consumption after 24h fasting. *Bdnf-e2*$^{-/-}$ mice, red; WT mice, blue. n=5~7/group.

Figure S7

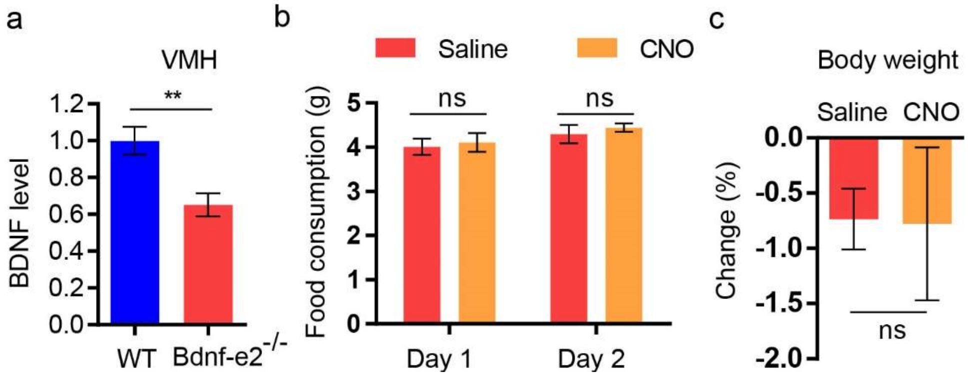

Figure S7: CNO treatment without AAV infection.
(a) Relative levels of BDNF protein in VMH of *Bdnf*-e2$^{-/-}$ and WT mice. **(b,c)** Food consumption (b) and change of body weight (c) after treatment of CNO or saline in *Bdnf*-e2$^{-/-}$ mice without AAV infection.

Figure S8

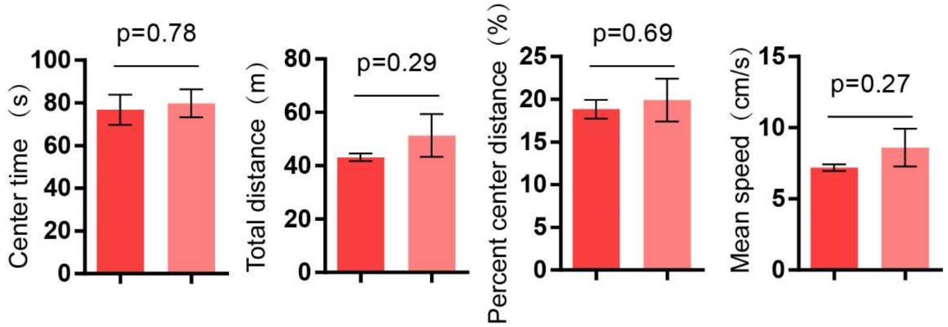

Figure S8: Effects of TrkB agonistic antibody injected into VMH of *Bdnf*-e2$^{-/-}$ mice on open field test.

Center time, total distance, percent center distance and mean speed during 10 minutes of open field test on the third day of *Bdnf*-e2$^{-/-}$ mice injected with TrkB-ago or IgG into VMH (n=4/group).
